# A Novel Hypertension Management Algorithm Guided by Hemodynamic Data

**DOI:** 10.1016/j.ekir.2021.11.029

**Published:** 2021-11-27

**Authors:** Barbara Greco, Yossi Chait, Brian H. Nathanson, Michael J. Germain

**Affiliations:** 1 Section of Nephrology, University of Massachusetts-Baystate Medical Center, Springfield, Massachusetts, USA; 2 Department of Mechanical Engineering, University of Massachusetts, Amherst, Massachusetts, USA; 3 OptiStatim, LLC, Massachusetts, USA; 4 Renal and Transplant Associates of New England, Springfield, Massachusetts, USA

**Keywords:** central blood pressure, hypertension, impedance cardiography

## Introduction

Hypertension affects approximately one-third of adult Americans (∼67 million). Only an estimated 43.7% of patients with hypertension achieve target blood pressure (BP).^1^ This number is significantly lower for patients with chronic kidney disease (CKD) and resistant hypertension.^2^ Current hypertension management guidelines permit a range of options regarding the selection of antihypertensive agents which contributes to variable practice patterns.^3^^,^^4^ Moreover, practitioners usually adopt suboptimal trial and error approaches when treating patients with hypertension. In addition, patients with hypertension do not respond uniformly to antihypertensive therapies because hypertension is a heterogeneous hemodynamic disorder.^5^

Bioimpedance or impedance cardiography (IC) is a novel tool in the management of hypertension with most of these studies using thoracic IC technology.^6^ Unfortunately, patients with CKD have been excluded from most of these reports. To address this gap, we undertook a pragmatic continuous quality improvement project in a real-world nephrology practice setting evaluating the use of hemodynamic parameters obtained using NICaS (a noninvasive cardiac system; Supplementary Appendix S1), a regional IC technology, to individualize the management of patients with hypertension and CKD.

## Results

A total of 93 patients were included in the analysis: 73 in the study group and 20 controls. Demographic characteristics did not differ significantly between the groups (Supplementary Table S1). The mean (SD) age was 60.5 (16.4) years for the study patients and 63.3 (10.7) years for the controls. Diabetes was present in 38.4% of the study patients and 30.0% of the controls, and approximately two-thirds had CKD stage ≥ 2. By design, the study patients had a significantly higher mean number of NICaS tests than the controls: 2.6 vs. 2.0, *P* < 0.001. For the study group, hemodynamic data obtained using NICaS were used in the selection and titration of antihypertensive medications according to a predetermined algorithm (Supplementary Table S2).

The hemodynamic profiles of the groups at baseline and final time points are illustrated in Supplementary Figure S1. At baseline, 41.1% of the study group patients had vasoconstricted physiology, 41.1% had mixed hemodynamics, and 17.8% were hyperdynamic. There were no significant differences in baseline hemodynamic profiles. At final analysis, 68.5% (50 of 73) of the study group patients achieved a normal physiological profile compared with 35.0% (7 of 20) of the controls (*P* = 0.006).

**Figure 1:**
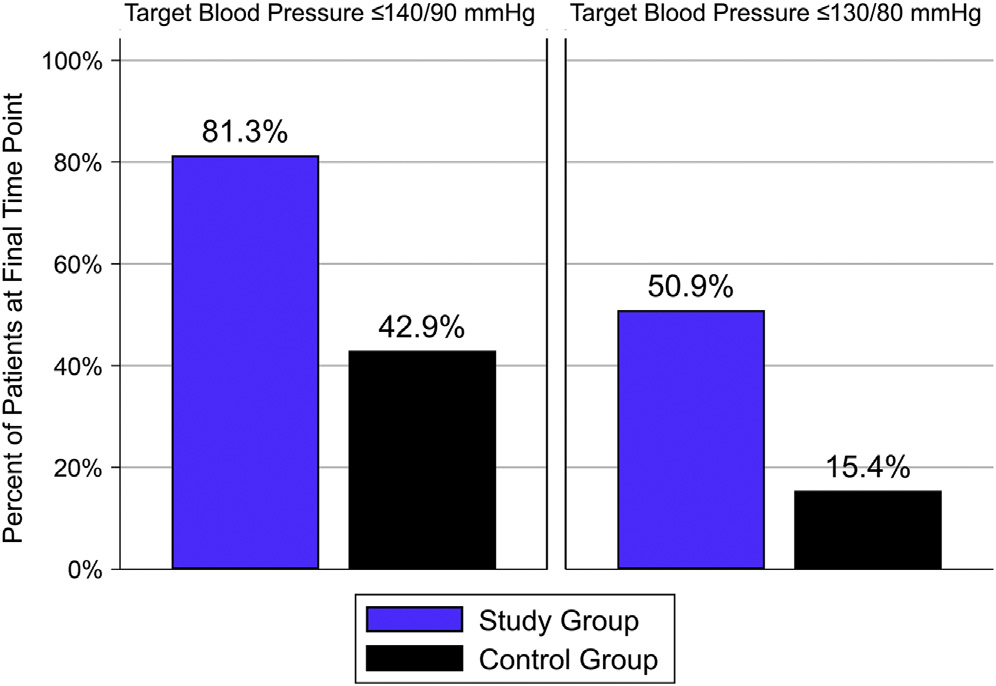
The graph illustrates the % of patients who met their respective hard or soft blood pressure goal at the final time point of the study period.

Table 1 reveals a comparison of baseline and final BP and hemodynamic parameters. Figure 1 illustrates the percentage of patients who met their respective BP goal at the final analysis. Given the “real world” nature of this project, BP values clinically close to the “hard” target of 130/80 mm Hg (e.g., 133/83 mm Hg) could be deemed acceptable with no change in treatment. Patients meeting these doctor-adjudicated situations were considered to meet their “soft target.” Mean baseline BP for both groups was statistically similar: 161.2/89.8 mm Hg (study) versus 163.4/89.8 mm Hg (control). There was a significantly greater drop in systolic, diastolic, and mean BP from baseline for the study patients than the controls. The study patients had a mean (SD) decrease in systolic BP of 24.2 (15.6) mm Hg compared with 14.5 (21.4) mm Hg in the controls (*P* = 0.025) along with a significantly greater mean (SD) decrease in diastolic BP of 12.8 (10.1) mm Hg versus 7.3 (9.7) mm Hg, *P* = 0.031 and mean arterial pressure of 16.4 (10.8) mm Hg versus 9.5 (12.7) mm Hg, *P* = 0.018. Significantly more patients in the study group met the hard target criteria: 57.53% versus 25.00%, *P* = 0.010, and for the soft target of 130/80 mm Hg at 50.88% versus 15.38% *P* = 0.029 (Supplementary Table S3). Compared with the controls, a greater percentage of patients without CKD achieved the target: 81.25% versus 28.57% (*P* = 0.026). Within-group vital signs and hemodynamic data at baseline and final time points are found in Supplementary Table S4. Changes in central hemodynamic parameters are reported in Supplementary Tables S5 and S6.

**Table 1:** Blood pressure and hemodynamic variables within the study and control groups at baseline and final time points

Variables, mean (SD) or *n* (%)	Study group at baseline *n* = 73	Study group at final point *n* = 73	*P* values comparing study group baseline with final points	Control group at baseline *n* = 20	Control group at final point *n* = 20	*P* values comparing control group baseline with final point
SBP	161.2 (15.6)	137.0 (15.4)	<0.001	163.4 (16.4)	148.9 (18.9)	0.007
DBP	89.8 (12.0)	77.0 (11.7)	**<**0.001	89.8 (11.6)	82.5 (18)	0.003
MAP	113.3 (9.9)	97.0 (11.0)	<0.001	113.9 (9.1)	104.4 (10.7)	0.003
HR	78.0 (13.0)	76.5 (11.2)	0.301	74.2 (17.8)	74.9 (12.4)	0.840
BMI	31.1 (7.8)	31.4 (8.1)	0.271	31.3 (5.3)	31.3 (5.3)	0.466
SI	39.0 (8.8)	41.9 (11.0)	0.020	38.2 (9.5)	39.7 (7.6)	0.466
CI	3.0 (0.9)	3.2 (1.0)	0.216	2.8 (0.8)	3.0 (0.7)	0.295
CPI	0.8 (0.2)	0.7 (0.2)	0.009	0.7 (0.2)	0.7 (0.2)	0.677
TPRI	3193.3 (863.1)	2612.3 (755.8)	<0.001	3549.8. (1151.9)	2972.3 (779.1)	0.002
Vasoconstricted	30 (41.1%)	7 (9.6%)	<0.001	9 (45.0%)	6 (30.0%)	0.453
Hyperdynamic	13 (17.8%)	6 (8.2%)	0.167	3 (15.0%)	3 (15.0%)	1.000
Mixed hemodynamic	30 (41.1%)	10 (13.7%)	<0.001	8 (40.0%)	4 (20.0%)	0.219
Normal hemodynamics	0 (0%)	50 (68.5%)	<0.001	0 (0%)	7 (35.0%)	0.016

BMI, body mass index; CI, cardiac index; CPI, cardiac power index; DBP, diastolic blood pressure; HR, heart rate; MAP, mean arterial pressure; SBP, systolic blood pressure; SI, stroke index; TPRI, total peripheral resistance.

The percentage of study group patients who had resistant hypertension, defined by uncontrolled BP despite 3 antihypertensives including a diuretic, was 13.7% versus 15.0% in the control group. There were no differences between the groups at study end regarding medication classes or number of antihypertensive medications (Supplementary Table S7). Nevertheless, significantly more patients in the study group compared with baseline were on select medications. There were no significant differences in the percentage of patients at baseline and final analysis in either group treated with thiazide or loop diuretics.

## Discussion

Previous studies suggested that management strategies incorporating hemodynamic data may be superior to standard care.S1–S3 Our findings confirm these results and reveal the feasibility and effectiveness of incorporating hemodynamic data derived from the validated^7^, ^8^, ^9^ NICaS bioimpedance system in the management of hypertension in patients with and without CKD in a clinical practice setting. The NICaS bioimpedance technology has greater accuracy and^9^ provides somewhat different hemodynamic information compared to thoracic IC because it measures blood flow and arterial resistance in the periphery rather than centrally. Neither technology accurately measures blood volume. The hemodynamic patterns observed here are notably heterogeneous.S4 Further research is needed to reveal whether normalization of hemodynamic parameters confers additional cardiovascular benefits beyond those of BP control.

We did not observe an increase in the use of thiazide or loop diuretics at the final period in contrast with the findings of Taler *et al.*S1 The difference may be partly explained by differences in the cohorts; our cohort had a smaller proportion of patients with resistant hypertension. Yet, given the significant percentage with CKD in our study, it is still surprising. It is possible that the assessment of total body water and effective volume status using the regional versus thoracic IC techniques provided conflicting data regarding fluid status. We observed a significant increase in the use of angiotensin receptor blockers in the treatment arm.S3 We also noted increased used of dihydropyridine calcium channel blockers, mineralocorticoid antagonists, selective beta-blockers and combination of nonselective beta-blockers, and alpha blockers between baseline and final analysis in patients managed according to the algorithm. The increase in use of mineralocorticoid antagonists may have offset the use of thiazides and loop diuretics.

There are several limitations in this study. First, because it was a continuous quality improvement project, intervals between visits were not standardized but instead reflect normal practice patterns. Next, the control group is small and our inferences were potentially underpowered though we observed statistically significant differences between groups in many key variables. This pragmatic project includes the possibility of bias based on physician practice styles and treatment effect. Another limitation is that the assessment of volume status using IC has not been validated in hypertensive cohorts. Therefore, it is possible that our definition of hypervolemia underestimated the percentage who may have responded to diuretic therapy. Finally, we did not exclude patients with congestive heart failure whose management is complicated by episodes of decompensation with more loop diuretics and/or holding renin angiotensin system inhibitors with increasing creatinine and/or potassium. Consequently, our findings may not be generalizable to this subpopulation of patients with hypertension.

In summary, this pragmatic continuous quality improvement program reveals the feasibility and effectiveness of a hemodynamically directed management algorithm to individualize hypertension management in a busy nephrology practice. This approach was associated with improved brachial and central BP control and normalization of cardiovascular hemodynamics in a cohort of patients with and without CKD. Although this study is hypothesis generating, the results suggest that hemodynamically guided hypertension management may result in more patients achieving target BP and improved physiology.

## Disclosure

BG and MJG report receiving funding, through their medical practice, from NiMed. All the other authors declared no competing interests.

## Supplementary Material

**Supplemental Methods.**

**Appendix S1.** Regional bioimpedance cardiography (NiCaS) measurements.

**Table S1.** Demographics of the study cohort.

**Table S2.** Algorithm for the management of hypertension by hemodynamic profile.

**Table S3.** In-target blood pressure results at baseline and final time points.

**Table S4.** Vital signs and hemodynamic data at baseline and final time points.

**Table S5.** Comparisons of central blood pressure and pulse wave analysis results within the study and control groups at baseline and final time points.

**Table S6.** Central blood pressure and pulse wave analysis at baseline and final time points.

**Table S7.** Antihypertensive medication classes within the study and control groups at baseline and final time points.

**Figure S1.** Hemodynamic profiles of the study and control groups at baseline and final time points. Red triangles, black circles, and blue squares represent means of SBP, MAP, and DBP for the entire group (irrespective of BP target), respectively.
