# Supplementary material for: A Novel Hypertension Management Algorithm Guided by Hemodynamic Data
Source: Kidney Int Rep. 2021 Nov 27;7(2):330–3. doi: 10.1016/j.ekir.2021.11.029 (PMC8820980; doi:10.1016/j.ekir.2021.11.029)
Supplement: Supplementary File (PDF) [file mmc1.pdf]

Supplemental Methods

This project was a prospective pragmatic continuous quality improvement initiative which took place in a large nephrology outpatient practice setting in the northeast United States between July of 2018 and April of 2019. Two nephrologists in the practice were trained on the interpretation of NICaS measurements and created a treatment algorithm based on the hemodynamic data. Patients were included in the program if they had been seen in the practice at least once and had uncontrolled BP (>140/90). Target BP was defined as ≤ 140/90 mmHg for patients without CKD, or ≤ 130/80 mmHg for patients with diabetes mellitus (DM) or CKD. All undertook measurements using NICaS at baseline and at final time point, with study patients undertaking NICaS at each office assessment. Blood pressures for all patients were obtained using the automated Sphygmacor technology (ATCOR Naperville, IL) which provides brachial BP as an average of three BP readings as well as central BP and pulse wave analysis (PWA). This was done with the patient seated for at least 5 minutes. This is considered the "gold standard" for office BP.

Patients under the care of the two trained nephrologists and who had at least two NICaS tests over a 4-month period constituted the Study Group. The Control group was comprised of patients under the care of other nephrologists in the practice and had at least two NICaS measurements; these patients were managed according to the discretion of their nephrologists who were made aware that the patient was in the program but had no access to their hemodynamic data. Inclusion of patients in this program received an institutional review board (IRB) waiver as it was an extension of practice in an American Society of Hypertension/American Heart Association Certified Hypertension Practice Center and a quality improvement project. (Quorum IRB Seattle, WA QR #: 33055). The project initially recruited Study (treatment) group participants due to expected additional visits per protocol consecutively,

then recruited control group patients that met inclusion criteria. We did not meet our target control group size due to insufficient patient interest during the fixed time period of the project.

For the Study group, hemodynamic data obtained using NICaS was used in the selection and titration of antihypertensive medications according to a pre-determined algorithm (see Supplemental Table 1; Key hemodynamic relations are given in Supplemental Table 2). Angiotensin Converting Enzyme inhibitor (ACEi) or angiotensin receptor blocker (ARB) was used in patients with clinical indications as per standard of care. Definitions of hemodynamic phenotypes were as follows:

- Vasoconstricted: if cardiac power index (CPI)<0.85 and total peripheral resistance (TPR) index (TPRI)>3000 and cardiac index (CI) <2.85
- Hyperdynamic: if CPI>0.85 and TPRI<3000 and CI>3.6
- Mixed hemodynamic: if hypertensive and neither vasoconstricted nor hyperdynamic.

Patients were considered hypovolemic if vasoconstricted with low total body water (TBW) and stroke index (SI)<30. Hypervolemia was defined as hyperdynamic physiology with high TBW and SI>30.

NICaS and Sphygmacor measurements were performed by medical assistants. The NICaS procedure is performed with the patient supine. NICaS utilizes regional IC which relies on passing an imperceptible alternating electrical current of 1.4 mA with a 30 kHz frequency through the arterial circulation via 2 pairs of sensors and records changes of electrical resistance of the arterial system by the same sensors. Sensors are placed on the left wrist above the radial pulse and the contralateral ankle above the posterior tibial pulse. This device provides accurate hemodynamic data including stroke volume (SV), heart rate (HR), cardiac output (CO), Cardiac

power (CP), Total peripheral resistance (TPR), and Total body water (TBW). SV is calculated by a proprietary algorithm based on changes in electrical resistance which are correlated to the ejection of blood from the left ventricle to the arterial system during the systolic phase. The hemodynamic data derived from this device have been validated in a number of studies. [7,8,9] Changes in BP parameters as well as hemodynamic phenotypes were analyzed along with changes made in the anti-hypertensive agents. Visit intervals varied over the study period consistent with normal practice patterns.

STATISTICAL METHODS

Descriptive statistics consisting of means and standard deviations for continuous variables and counts and frequencies for categorical variables were calculated for all clinical and demographic variables. We used the Student's t-test to compare means between the Study and Control groups. To compare proportions, we used the Chi-Square test unless a cell count was < 5, wherein we used the Fisher's exact test. To compare baseline to final values within each group, we used the paired t-test for continuous variables and McNemar's test for binary variables and derived p-values using exact statistics when a cell count was < 5. For comparisons within groups (eg, for the same patients at baseline and final analysis), continuous variables were compared using the paired t-test and categorical variables were compared using McNemar's test. We did not adjust for differences in baseline characteristics between groups but focused on paired analyses (ie, patients serving as their own control) when possible. Missing data were rare and due to a small number of patients dropping out of the analysis. We assumed data was missing at random and consequently performed no imputation present a complete case analysis. All two-sided p-values

<0.05 were considered statistically significant. All analyses were done using Stata/MP 15.1 for Windows (StataCorp LLC, College Station, TX).

We reported BP results in terms of hard targets as well as soft targets. We defined "soft target" as BPs that in addition to presentation are sufficiently close to target such that no further medication adjustment was made by the nephrologists. For example, a patient could have a blood pressure of 133/80 mmHg and a hard target of 130/80 mmHg. Thus, the "soft target" analysis is sensitivity analysis reflecting "real world" practice. We adjudicated these cases individually and found that in the majority of these patients, Sphygmacor BP was at or within 4 mmHg of target. There were 18 such patients in the cohort.

Supplemental Appendix s1: Regional Bioimpedance Cardiography (NiCaS) Measurements

The device used to measure hemodynamics, NICaS or Noninvasive Cardiac System, is based on bioimpedance technology. Stroke Volume is measured by applying alternating electrical current of 1.4 mA with a 30 kHz frequency through the patient body via two pairs of tetrapolar sensors, one pair placed on the wrist of the hand without the arteriovenous fistula or graft above the radial pulse and the other pair on the contralateral ankle above the posterior tibial arterial pulse. SV is calculated by Frinerman's formula. [s5-s6]

$$\text{Stroke Volume} = (dR/R) \times (\rho) \times (L^2/Ri) \times (\alpha + \beta)/\beta \times (KW) \times (HF) \ \text{(ml)}$$

where dR is impedance change of the arterial system as a result of arterial system expansion during the systole, R is basal resistance (ohms), ρ is blood electrical resistance (ohms), L is patient's height (meter), Ri is corrected basal resistance according to gender and age (ohms), KW is the

correction of weight according to ideal values, HF is a hydration factor which takes into account the body water composition, α+ β is the ECG R-R wave interval, and β is the diastolic time interval. SV is automatically calculated every 20 second and the average of three measurements obtained consecutively during 60 seconds of monitoring (see figure below).

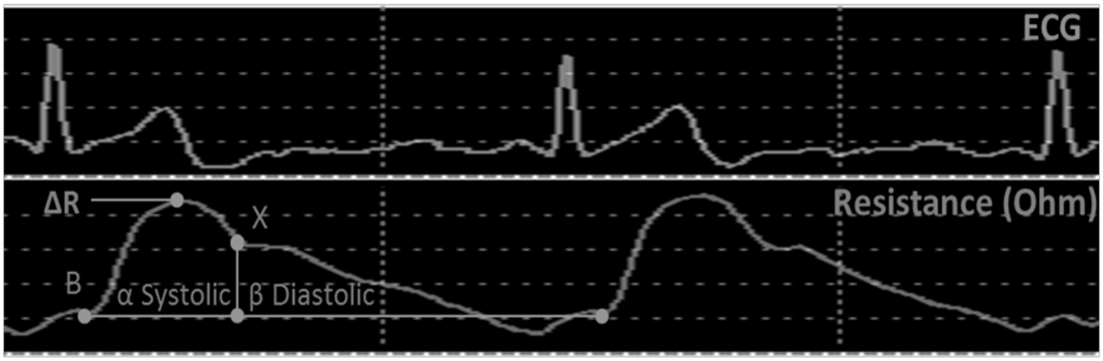

ΔR: Max. Change of resistance B: Aortic valve open X: Aortic valve close

Sample NiCaS measurement panel

Hemodynamic Relations

**Parameter**	**Symbol**	**Definition**	**Normal Range**	**Derivation Formula**
Heart rate	HR	Number heart bpm	60-90 bpm	Measurement of R-R
Stroke volume	SV	Amount of blood pumped by LV per heart beat	35-64 ml/min	S≅ ΔR/R
Stroke index	SI	Amount of blood pumped by the LV per heartbeat normalized to bsa	35-64 ml/min/m^2	SI=SV/BSA
Cardiac output	CO	Amount of blood pumped by LV per min	2.5-4 L/min	CO=HRxSVx1000
Cardiac index	CI	Amount of blood pumped by the LV per min normalized to bsa	2.5-4 L/min/m^2	CI=CO/BSA

Cardiac power index	CPI	An indication of cardiac contractility	0.45-0.85 watts/m^2	CPI=COxMAPx0.0022
Total peripheral resistance	TPR	Resistance to flow in arterial system	1600-3000 dyn x sec/cm^5	TPR= 80xMAP/CO
Total peripheral resistance index	TPRI	TPR normalized to bsa	TPR/m^2	TPRI=80xMAP/CI
Total body water	TBW	Amount of fluids	Individually calculated per gender and BMI	TBW=Ht^2/R

Abbreviations: bpm: beats per minute; min: minute; LV: left ventricle; bsa: body surface area; ml: milliliters; m: meters; sec: seconds; cm: centimeter; BMI: body mass index; Ht: height

Supplemental Table s1: Demographics of the Study Cohort

Variable	Study Group N = 73	Control Group N = 20	P-value
Age at baseline (Mean, SD)	60.5 (16.4)	63.3 (10.7)	0.490
Female	36 (49.3%)	13 (65.0%)	0.213
Race			
White	50 (68.5%)	14 (70.0%)	0.132
Black	9 (12.3%)	0 (0%)	
Hispanic	10 (13.7%)	6 (30%)	
Asian/Other/Unknown	4 (5.5%)	0 (0%)	
CKD Level at baseline			
0	19 (26.0%)	7 (35.0%)	0.139
1	9 (12.3%)	0 (0%)	
2	22 (30.1%)	3 (15.0%)	
3	18 (24.7%)	7 (35.0%)	
4	3 (4.1%)	3 (15.0%)	
5	2 (2.7%)	0 (0%)	
Diabetes Type at baseline			
None	45 (61.6%)	14 (70.0%)	0.558
1	2 (2.7%)	1 (5.0%)	
2	26 (35.6%)	5 (25.0%)	
Transplant Present at baseline	9 (12.3%)	1 (5.0%)	0.684
Total Number of NICAS Measurements; Mean (SD)	2.6 (0.7)	2.0 (0)	<0.001
Total Number of MD Office Visits; Mean (SD)	3.2 (1.1)	2.9 (1.1)	0.278

Supplemental Table s2 Algorithm for the Management of Hypertension by Hemodynamic Profile

<u>Actions:</u>

Vasoconstricted

- Vasodilators (afterload reduction): add/increase ACEI or ARB or alpha-blockers or CCB w/o negative inotropic effect. Consider direct vasodilators in resistant cases. Consider other variables, such as creatinine, potassium, proteinuria, drug allergy, prostatic hypertrophy, in choosing the appropriate type of vasodilator.
- Diuretics: reduce/stop.
- Beta-blockers: reduce/stop.
- If vasoconstricted and high arterial stiffness (via SphygmaCor):
  - Vasodilators (afterload reduction): RAAS inhibitors appear superior to other classes. Consider switch to bystolic from other beta-blockers. ARBs seemingly have a beneficial effect on arterial stiffness, with the caveat that results are conflicting.

Hyperdynamic

- Vasodilators (afterload reduction): reduce/stop.
- Beta-blockers: add/increase if high HR (>80).
- Diuretics: add/increase if hypervolemic.
  - Add diuretic and/or CCB with negative inotropic effect if normal HR (80) and high SI (>50):

Mixed

- Vasodilators (afterload reduction): add/increase ACEI or ARB or alpha-blockers or CCB. Consider direct vasodilators in resistant cases. Consider other variables, such as creatinine, potassium, proteinuria, drug allergy, prostatic hypertrophy, in choosing the appropriate type of vasodilator.
- Beta-blockers: add/increase if high HR (>80).
- Diuretics: add/increase if hypervolemic.
  - Add diuretic and/or CCB with negative inotropic effect if high SI (>50).

CHF Considerations

In patients with history of CHF, in addition to above use diuretics as follows:

If low SI (<30) and low TBW or (normal TBW + no edema):

- Diuretics: reduce/stop.

If low SI (<30) and normal/high TBW and pulmonary edema (shortness of breath):

- Diuretics: add/increase; Monitor closely for decreased SI.

Abbreviations: ACE I: angiotensin converting enzyme inhibitor; ARB: angiotensin receptor blocker; CCB: calcium channel blocker; HR: heart rate; SI: stroke index; TBW: total body water' CHF: congestive heart failure

Supplemental Table s3: In Target Blood Pressure Results at Baseline and Final Time Points

**Results for Patients with 130/80 Hard Target Blood Pressure (BP)**	**Study Group N = 57**	**Control Group N =13**	**P-value**
Number of patients in the 130/80 hard target BP group meeting this criterion at baseline	0/57 (0%)	0/13 (0%)	1.000
Number of patients in the 130/80 hard target BP group meeting this criterion at final time point (≤ for both SBP and DBP)	12/57 (21.05%)	2/13 (15.38%)	1.000
Number of patients in the 130/80 hard target BP group meeting this criterion at final time point (≤ for both SBP and DBP) or meeting the "soft target" criterion	29/57 (50.88%)	2/13 (15.38%)	0.029
**Results for Patients with 140/90 Hard Target Blood Pressure (BP)**	**Study Group N = 16**	**Control Group N =7**	**P-value**
Number of patients in the 140/90 hard target BP group meeting this criterion at baseline	0/16 (0%)	0/7 (0%)	1.000
Number of patients in the 140/90 hard target BP group meeting this criterion at final time point (≤ for both SBP and DBP)	13/16 (81.25%)	2/7 (28.57%)	0.026
Number of patients in the 140/90 hard target BP group meeting this criterion at final time point (≤ for both SBP and DBP) or meeting the "soft target" criterion	13/16 (81.25%)	3/7 (42.86%)	0.137
**Results as Final Time Point for All Patients**	**Study Group N = 73**	**Control Group N =20**	**P-value**
Number of patients meeting their corresponding hard targets	25/73 (34.25%)	4/20 (25.00%)	0.283
Number of patients meeting their corresponding "soft targets"	42/73 (57.53%)	5/20 (25.00%)	0.010

Supplemental Table s4: Vital Signs and Hemodynamic Data at Baseline and Final Time Points

**Baseline Characteristics (Mean (SD) or N (%))**	**Study Group N =73**	**Control Group N = 20**	**P-value**
SBP	161.2 (15.6)	163.4(16.4)	0.588
DBP	89.8 (12.0)	89.8 (11.6)	0.977
MAP	113.3 (9.9)	113.9 (9.1)	0.815
HR	78.0 (13.0)	74.2 (17.8)	0.287
BMI	31.1 (7.8)	31.3 (5.3)	0.907
SI	39.0 (8.8)	38.2 (9.5)	0.735
CI	3.0 (0.9)	2.8 (0.8)	0.261
CPI	0.8 (0.2)	0.7 (0.2)	0.276
TPRI	3193.3 (863.1)	3549.8. (1151.9)	0.133
**Final Time Point Characteristics (Mean (SD) or N (%))**	**Study Group N =73**	**Control Group N = 20**	**P-value**
SBP	137.0 (15.4)	148.9 (18.9)	0.004
DBP	77.0 (11.7)	82.5 (18)	0.070
MAP	97.0 (11.0)	104.4 (10.7)	0.009
HR	76.5 (11.2)	74.9 (12.4)	0.572
BMI	31.4 (8.1)	31.3 (5.3)	0.961
SI	41.9 (11.0)	39.7 (7.6)	0.409
CI	3.2 (1.0)	3.0 (0.7)	0.286
CPI	0.7 (0.2)	0.7 (0.2)	0.984
TPRI	2612.3 (755.8)	2972.3 (779.1)	0.064

Supplemental Table s5: Comparisons of Central Blood Pressure and Pulse Wave Analysis Results within Study and Control Groups at Baseline and Final Time Points

Variable (Mean (SD) or N (%))	Study Group at Baseline N =73	Study Group at Final Point N =73	P-value comparing Study group Baseline to Final Point	Control Group at Baseline N = 20	Control Group at Final Point N = 20	P-value comparing Control Group Baseline to Final Point
Central Systolic Pressure	140.7 (21.4)	116.4 (31.1)	<0.001	144.8 (14.8)	132.2 (16.3)	0.008
Central Diastolic Pressure	90.3 (15.9)	75.2 (21.3)	<0.001	91.3 (11.7)	83.8 (11.9)	0.002
Central Pulse Pressure	50.3 (16.7)	41.2 (16.8)	<0.001	53.5 (18.8)	48.4 (17.9)	0.129
Central Augmentation Pressure	11.8 (8.8)	9.8 (10.3)	0.107	10.9 (10.5)	11.3 (10.4)	0.819
Central Augmentation Index	21.3 (12.1)	20.0 (20.9)	0.628	18.2 (14.4)	20.2 (16.2)	0.440
Forward Pulse Height	37.5 (10.7)	30.7 (11.5)	<0.001	37.5 (8.6)	35.1 (10.3)	0.192
Reflected Pulse Height	21.5 (7.2)	17.6 (7.2)	<0.001	21.5 (6.3)	20.1 (7.0)	0.198
Reflection Magnitude %	56.8 (11.8)	54.5 (17.7)	0.298	57.1 (9.7)	57.0 (10.0)	0.923
PPA	139.6 (20.5)	133.7 (36.0)	0.209	141.5 (15.0)	140.1 (12.1)	0.628

Supplemental Table s6: Central Blood Pressure and Pulse Wave Analysis at Baseline and Final Time Points

**Variable at Baseline**	**Study Group N =73**	**Control Group N = 20**	**P-value**
Central Systolic Pressure	140.7 (21.4)	144.8 (14.8)	0.413
Central Diastolic Pressure	90.3 (15.9)	91.3 (11.7)	0.790
Central Pulse Pressure	50.3 (16.7)	53.5 (18.8)	0.465
Central Augmentation Pressure	11.8 (8.8)	10.9 (10.5)	0.704
Central Augmentation Index	21.3 (12.1)	18.2 (14.4)	0.335
Forward Pulse Height	37.5 (10.7)	37.5 (8.6)	0.985
Reflected Pulse Height	21.5 (7.2)	21.5 (6.3)	0.979
Reflection Magnitude %	56.8 (11.8)	57.1 (9.7)	0.906
PPA	139.6 (20.5)	141.5 (15.0)	0.707
**Variable at the Final Time Point**	**Study Group N =73**	**Control Group N = 20**	**P-value**
Central Systolic Pressure	116.4 (31.1)	132.2 (16.3)	0.031
Central Diastolic Pressure	75.2 (21.3)	83.8 (11.9)	0.085
Central Pulse Pressure	41.2 (16.8)	48.4 (17.9)	0.100
Central Augmentation Pressure	9.8 (10.3)	11.3 (10.4)	0.589
Central Augmentation Index	20.0 (20.9)	20.2 (16.2)	0.971
Forward Pulse Height	30.7 (11.5)	35.1 (10.3)	0.123
Reflected Pulse Height	17.6 (7.2)	20.1 (7.0)	0.180
Reflection Magnitude %	54.5 (17.7)	57.0 (10.0)	0.554
PPA	133.7 (36.0)	140.1 (12.1)	0.438

Supplemental Table s7: Anti-Hypertensive Medication Classes within Study and Control Groups at Baseline and Final Time Points

**Medication Classes: N (%)**	**Study Group at Baseline N =73**	**Study Group at Final Point N =73**	**P-value comparing Study group Baseline to Final Point**	**Control Group at Baseline N = 20**	**Control Group at Final Point N = 20**	**P-value comparing Control Group Baseline to Final Point**
ACE Inhibitor	8 (11.0%)	14 (19.2%)	0.031	7 (35.0%)	7 (35.0%)	1.000
ARBs	15 (20.5%)	25 (34.2%)	0.013	3 (15.0%)	3 (15.0%)	1.000
CCB: dihydropyridine	24 (32.9%)	52 (71.2%)	<0.001	9 (45.0%)	12 (60.0%)	0.375
Direct Acting Vasopressor	2 (2.7%)	4 (5.5%)	0.625	3 (15.0%)	3 (15.0%)	1.000
CCB: non dihydropyridine	1 (1.4%)	1 (1.4%)	1.000	1 (5.0%)	1 (5.0%)	1.000
Loop Diuretic	3 (4.1%)	6 (8.2%)	0.250	1 (5.0%)	1 (5.0%)	1.000
Thiazide Diuretic	12 (16.4%)	19 (26.0%)	0.119	2 (10.0%)	3 (15.0%)	1.000
Aldosterone Receptor or ENaC blocker	5 (6.8%)	14 (19.2%)	0.004	1 (5%)	2 (10.0%)	1.000
Beta Blocker-Selective	4 (5.5%)	14 (19.2%)	0.006	3 (15.0%)	3 (15.0%)	1.000
Beta Blocker-Non-Selective + Alpha	7 (9.6%)	20 (27.4%)	0.001	3 (15.0%)	2 (10%)	1.000
Beta Blocker-Non-Selective	0 (0%)	0 (0%)	1.000	0 (0%)	0 (0%)	1.000
Alpha-1 Blocker	1 (1.4%)	2 (2.7%)	1.000	1 (5.0%)	2 (10.0%)	1.000
Central Alpha Blockers	3 (4.1%)	6 (8.2%)	0.375	2 (10%)	1 (5.0%)	1.000
Total Number of Drugs from the list above; Mean (SD)	1.2 (1.3)	2.4 (1.2)	<0.001	1.8 (1.1)	2.0 (1.0)	0.297
Resistant Hypertension	10 (13.7%)	10 (13.7%)	1.000	3 (15.0%)	1 (5.0%)	0.500

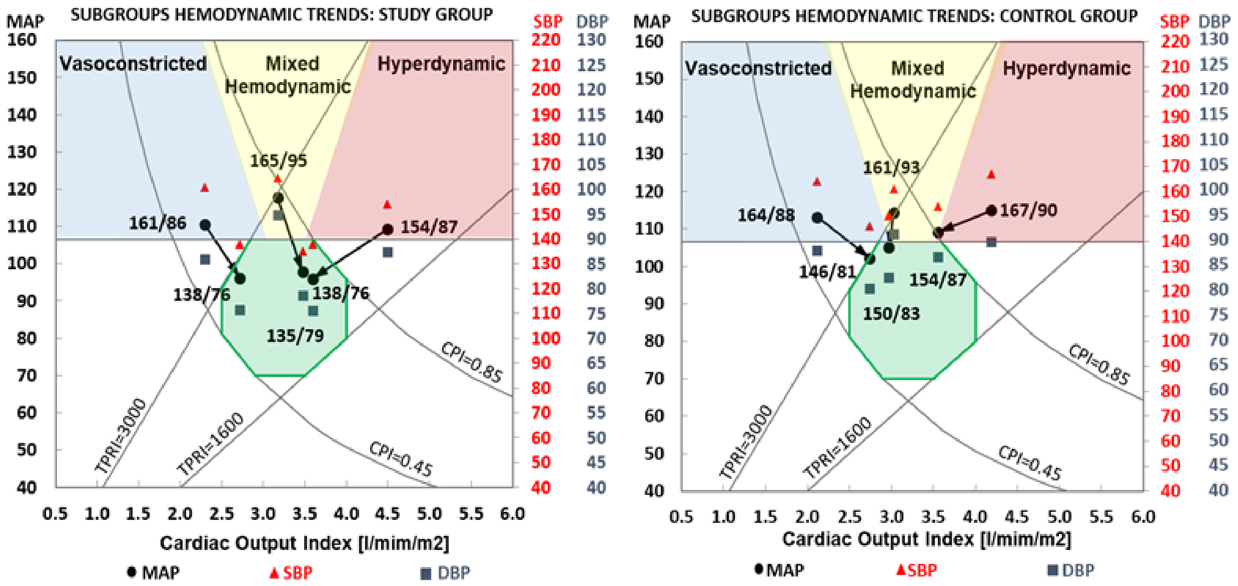

Supplemental Figure s1. Hemodynamic profiles of study and control groups at baseline and final time points. Red triangles, black circles, and blue squares reresent means of SBP, MAP, and DBP for the entire group (irrespectibe of BP target).

Supplementary References

s1. Taler S, Textor S, and Augustino J. Resistant Hypertension. Hypertension; 2002 May 39(5): 982-988.

s2. Sharman D, Gomes C, and Rutherford J. Improvement in Blood Pressure Control with Impedance Cardiography-Guided Pharmacologic Decision Making. Cong Heart Fail. 2004; 10: 54-58.

s3. Smith R, Levy P and Ferrario C. Value of Noninvasive Hemodynamics to Achieve Blood Pressure Control in Hypertensive Patients. Hypertension. 2006; 47: 771-777.

s4. Mahajian S, Gu J, Lu Y, Khera R, Spatz, E, Zhang M, Sun N, Zheng X, Zhao H, Lu H, Ma Z, Krumholz H. Hemodynamic phenotypes of hypertension based on cardiac output and systemic vascular resistance. Am. J. Med. 2020; 133(4): e127-e135.

s5.Cotter G, Moshkovitz Y, Kaluski E, et al. Accurate, non-invasive, continuous monitoring of cardiac output by whole body electrical bio-impedance. Chest. 2004; 125(4):1431-1440, PMID: 15078756.

s6.Leitman L, Sucher E, Kaluski E, et al. Noninvasive measurement of cardiac output by whole-body bio-impedance during dobutamine stress echocardiography: Clinical implications in patients with left ventricular dysfunction and ischaemia. European J Heart Fail. 2006;8(2):136-140, PMID: 16199201.
